# The new wave: time to bring EEG to the emergency department

**DOI:** 10.1186/1865-1380-4-36

**Published:** 2011-06-24

**Authors:** Samah G Abdel Baki, Ahmet Omurtag, André A Fenton, Shahriar Zehtabchi

**Affiliations:** 1 Bio-Signal Group Corporation, 760 Parkside Avenue, Brooklyn, NY, 11226-1508, USA; 2 Department of Emergency Medicine, State University of New York, Downstate Medical Center, Box 1228, Brooklyn, NY 11203, USA; 3 Center for Neural Science, New York University, New York, NY, USA; 4 The Robert F. Furchgott Center for Neural and Behavioral Science, State University of New York, Downstate Medical Center, Brooklyn, NY, USA

## Abstract

Emergency electroencephalography (EEG) is indicated in the diagnosis and management of non-convulsive status epilepticus (NCSE) underlying an alteration in the level of consciousness. NCSE is a frequent, treatable, and under-diagnosed entity that can result in neurological injury. This justifies the need for EEG availability in the emergency department (ED). There is now emerging evidence for the potential benefits of EEG monitoring in various acute conditions commonly encountered in the ED, including convulsive status after treatment, breakthrough seizures in chronic epilepsy patients who are otherwise controlled, acute head trauma, and pseudo seizures. However, attempts to allow for routine EEG monitoring in the ED face numerous obstacles. The main hurdles to an optimized use of EEG in the ED are lack of space, the high cost of EEG machines, difficulty of finding time, as well as the expertise needed to apply electrodes, use the machines, and interpret the recordings. We reviewed the necessity for EEGs in the ED, and to meet the need, we envision a product that is comprised of an inexpensive single-use kit used to wirelessly collect and send EEG data to a local and/or remote neurologist and obtain an interpretation for managing an ED patient.

## Introduction

Abundant literature has been accumulated during the last decade to characterize a well-defined, routine use of EEG in emergency departments (EDs) [1]. Routine use of EEGs in acute settings may advance patient care in certain neurological scenarios such as acute alteration of mental status (AMS) and severe traumatic brain injury (sTBI) [2-5]. In such clinical scenarios, access to cerebral function is often hindered by an unrevealing bedside physical exam in obtunded or deeply sedated subjects [6,7]. Since the initial call by Jordan (1995) [8] for a major monitoring system able to continuously evaluate cerebral functions in critically ill patients, several studies have aimed to characterize the role of the EEG in various clinical contexts, including the emergency department (ED). Taking in all the recent calls for the need for an emergency EEG system (eEEG), this article will propose a system compatible with ED use, and capable of enhancing the diagnosis and management of various neurological emergencies. First, we will briefly review the potential clinical impact of EEG availability in the ED by introducing data on acute entities commonly encountered in emergency settings with findings requiring the need for eEEG accessibility. Second, we will further expound on the notion of routine eEEG availability by unfolding the components of our proposed eEEG system. Lastly, we conclude by emphasizing the impact of eEEG on patient care and outcome.

### eEEG and non-convulsive status epilepticus

Non-convulsive status epilepticus (NCSE) was shown to occur in more than a third of patients with unexplained AMS [1]. NCSE may present a diagnostic challenge when an EEG is unavailable in the ED, which is often the case [9]. The lack of overt, tonic-clonic activity and the difficulties in identifying behavioral changes from baseline necessitate the presence of an EEG for confirming seizure activity. Early and recent studies done in the ED and the intensive care unit (ICU) have reported significant delays in the diagnosis of NCSE, especially when subtle alterations were attributed to other etiologies [10-12]. Apart from the wide range of behavioral manifestations occurring in NCSE that justify the need for routine EEG availability, NCSE may also include various ictal morphologies that are difficult to interpret in emergency settings [9]. The literature on EEG features in NCSE includes a spectrum of "read-outs" that could coexist in other entities, making distinction and consequent ictal identification more difficult. Perhaps the most notorious example would be the appearance of high-frequency triphasic waves in both hepatic encephalopathy and NCSE. The above calls for a reconsideration of the interpretation of emergent EEGs. In such particularly common scenarios of problematic judgments of EEG manifestations, a case management system is needed to allow a remote epileptologist to review eEEGs recorded in acute settings, such as the ED where an epileptologist is typically not present. Another feature of NCSE that argues for the importance of notifying a neurologist is treatment. Even when a diagnosis of NCSE can be made, treatment and its potential adverse events may present challenges to acute care/ED physicians. Furthermore, NCSE is more common in elderly patients, thus raising the possibility of a greater risk of systemic complications of antiepileptics [13,14].

The above challenges call for the use of a special emergency EEG system comprised of a single kit with all the components needed for rapidly collecting and wirelessly allowing EEG data to be shared by a local and/or remote neurologist for managing ED patients. We will describe the components of the eEEG system we envision after stating other commonly encountered acute entities that would benefit from such an application. Table 1 summarizes the diagnostic challenges of certain neurological entities and their benefit from EEG incorporation.

**Table 1:** Diagnostic challenges of neurological entities in the emergency setting and the benefits from EEG incorporation

I. Non-Convulsive Status Epilepticus (NCSE)
i. frequent unavailability of an EEG apparatus for a prompt identification of NCSE.

ii. variety of clinical manifestations including the wide spectrum of behavioral presentations.

iii. the differential diagnosis of altered mental status is vast and might consequently lead to a significant under-diagnosis of NCSE.

iv. even when an EEG device is available, EEG ictal identification of the variable EEG morphologies encountered in NCSE might require expert identification and interpretation.

v. unavailability of a neurologist to give an emergent interpretation.

II. Generalized Convulsive Status Epilepticus (GCSE)

i. high correlation with various acute brain injuries.

ii. NCSE might predominate after control of GCSE.

iii. specific EEG patterns after control of convulsions are correlated with prognosis.

III. Breakthrough Seizures

i. identification of underlying cause of seizure exacerbation.

ii. management of antiepileptic drug regimen.

IV. Severe Traumatic Brain Injury (sTBI)

i. "Pharmacologically" paralyzed patient where cerebral function cannot be strictly assessed clinically.

ii. management of neurological insults that could be delayed in appearing and thus raising the risk of irreversible cerebral damage.

iii. administration of various sedatives/analgesics that carry a high risk of sedation.

iv. evaluation of a consequent cerebral dysfunction that is paralleled by various extra cerebral defects.

### eEEG and convulsive status epilepticus

Generalized convulsive status epilepticus (GCSE) is a neurological emergency that carries a mortality risk of 7-39% and is associated with life-threatening sequelae if not managed in a timely manner [15-17]. As outlined by DeLorenzo et al. 1992 [18], more than 50% of reported GCSE cases result from various acute brain injuries. Therefore, CSE is an entity that is highly correlated with various neurological emergencies and deserves prompt early management. Clinical manifestations of CSE are often easily recognized when witnessed during the tonic-clonic episodes. Yet, after the control of such overt symptoms of GCSE, NCSE might predominate and result in persistent obtundation. This is evidenced by various studies reporting patients with GCSE who continued to have non-convulsive seizures (NCS) after cessation of convulsions [19,20].

Evaluating cerebral function after control of clinical CSE via EEG has changed our opinion regarding the assessment of outcome and treatment. Specific EEG patterns recorded after control of convulsions were shown to be significantly correlated with prognosis. In the study by Jaityl et al. [21], the presence of periodic lateralizing epileptiform discharges (PLEDs) was a functional predictor of a high mortality rate, whereas EEG normalization after CSE was correlated with a good outcome. Therefore, this indicates that EEG monitoring after clinical control of GCSE serves as a prognostic indicator, and clinical evidence argues for its availability in emergency settings.

### eEEG and breakthrough seizures

Beyond the well-defined need for EEG availability in the diagnosis and management of unexplained alterations in mental status, other acute entities encountered in the ED might benefit from this availability. Up to 30% of patients treated with antiepileptics continue to experience breakthrough seizures and often present to an emergency department [22,23]. This puts substantial demands on the ED physician at various levels, including (1) identification of the underlying cause of seizure exacerbation in an otherwise controlled patient and (2) management of the antiepileptic drug regimen.

Clinical management decisions, especially when adjusting regimens of antiepileptics, entail that an ED physician coordinates with a consulting neurologist. EEG availability with software that allows the access of a consulting neurologist in acute care settings can facilitate communication and provide a prompt course of action regarding drug adjustment. A sub-therapeutic level of antiepileptic medication most commonly causes breakthrough seizures [24]. Unlike the well-established therapeutic ranges for older antiepileptics, newer ones have a less defined therapeutic level, and clinical control of seizures is the rule of management. This further argues for the importance of equipping EDs with a system that allows for remote neurology consultation upon acquisition of an EEG, particularly when adjustment of a drug regimen is required [25,26].

### eEEG and severe traumatic brain injury

In sTBI patients, early institution of sedatives and analgesics for the maintenance of cerebral perfusion, control of agitation, and airway protection commonly results in a "pharmacologically" paralyzed patient [27]. The use of EEG in this scenario is beneficial in replacing an uninformative neurological bedside examination, and monitoring cortical activity and reactivity to drug administration. sTBI is heterogeneous and might result in various neurological sequelae including (1) elevated intracerebral pressure that could effectively lower cerebral perfusion and (2) intracerebral hemorrhages. The above consequences of sTBI could be delayed in emerging and be unnoticed by early cerebral imaging, thus justifying the need for a continuous neurophysiological monitor, for which EEG is appropriate [28,29].

Another reported consequence of sTBI that further justifies the need for EEG availability is NCSE [2]. Identification and interpretation of NCSE is further challenged in this context because of the common co-existence of extra-cerebral effects induced by trauma. Those extra-cerebral factors include trauma-induced cranial defects that could result in the production of EEG artifacts, and thus require accurate identification and interpretation.

The above observations should be considered as potential indications for the use of EEG monitoring in EDs not only for detecting epileptiform activity in a predisposed brain, but also for monitoring impending neuropathological consequences.

### eEEG and pseudononepileptic seizures

The incidence of pseudo-seizures (PS) is high, between 1.4 and 4 per 100,000 [30,31]. A major subcategory of these patients presents to EDs with pseudo-status epilepticus (PSt), an entity that puts patients at a high risk of iatrogenic harms comprised of unnecessary intravenous medications and ventilatory support for airway protection [32,33]. Unfortunately, the diagnosis of PS and PSt cannot be established in the ED and requires long-term inpatient EEG/video monitoring. A strong clinical suspicion usually precedes hospital admission and is crucial when observed during an attack by ED medical personnel [34,35].

Despite the established role of prolonged EEG/video in diagnosing PS, identification of suggestive features is still important for further diagnostic monitoring. The use of EEG during the paroxysmal episode may help in providing ED personnel with an early provisional diagnosis, which could determine further tests needed for a definitive diagnosis. Recent studies have highlighted the importance of suggestive features in raising clinical suspicion of a nonepileptic etiology during initial assessment [36,37]. Documenting a negative interictal EEG in the ED might enhance clinical suspicion and thereby preclude the need for inpatient monitoring.

It is worth noting that certain types of epileptic seizures such as frontal lobe seizures may be mistakenly diagnosed as psychogenic [38]. Frontal lobe seizures are initially distinguished from nonepileptic events through various features, including suggestive clinically bizarre movements, resistance to physical examination, as well as other historical features such as resistance to anti- epileptic drugs (AEDs). This further signifies the importance of preliminary suggestive features, which in turn may help in increasing or decreasing the index of suspicion in patients presenting with seizure-like symptomatology. One major study reported that subjects with two seizure-like events a week, which have shown resistance to at least two (AEDs), and who have had at least two EEGs without epileptiform anomalies have a more than 80% chance of having a nonepileptic seizure of psychogenic origin [39]. Thus, in many respects, the "certification" of a negative EEG might increase the diagnostic yield of other clinical/historical features in an acute setting where access to video/EEG is restricted.

### The eEEG system we would like to have

While there are many EEG recording systems and accessories in the market place, the eEEG system we envision must operate in the ED, which presents a unique set of challenges to obtaining a rapid EEG interpretation in less than ideal conditions.

**Figure 1:**
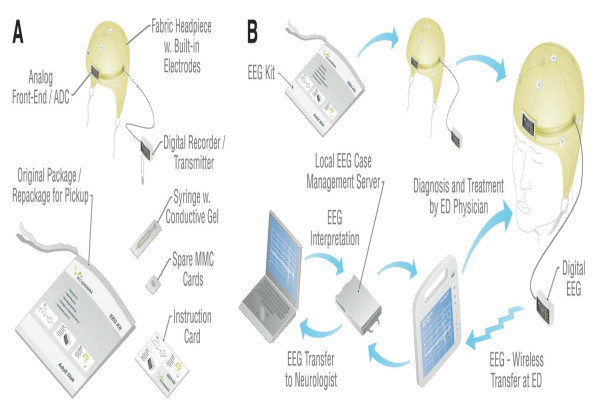
**Everything necessary to rapidly record and interpret the EEG in the ED**. (**a**) **The EEG-kit**: A sealed bag contains EEG kit components (electro-cap with integrated electrodes, analog front-end and analog-to-digital convertor electronics; a plug-in digital EEG transmitter and battery module; sterile electrode gel and applicator; operating instructions). (**b**) **The eEEG system**: Plugging in the transmitter/battery module activates electrode impedance testing to determine appropriate conductive contact to the scalp and give correcting feedback. Patient data are entered using the bar-code reader and keypad on the medical tablet PC. Once recording is initiated, EEG is wirelessly transmitted to the medical tablet for display and then to a case management server. While this is not a feature of the proposed system, the server will also perform real-time automatic seizure detection, setting EEGs with seizure abnormalities to high priority for review by one or more remote neurologists. Via e-mail and electronic instant messaging, the case management software notifies a network of board-certified neurologists who are available to read the EEGs using standard computers of their choosing. The responding neurologist logs in to access the EEG for review and provides a written interpretation. The interpretation is sent back to the ED physician to guide patient care and management. The EEG kit components are discarded and sent out for refurbishment.

We present a schematic eEEG system in Figure 1 and describe the functionality of the components. In conceiving the system we recognized that the basic requirements of the ED scenario can be met today by harnessing the current state-of-the-art EEG technology and knowledge from both research and clinical environments. The eEEG system we would like to have in the ED is summarized as follows: (1) a microEEG (low-noise, high common-mode rejection ratio, narrow window for noise entry) (2) an eEEG-kit: The product we envision in widespread use is an eEEG comprised of an inexpensive single-use "EEG-kit" with disposable/refurbishable components used to collect the EEG all gathered in a sealed plastic bag, and software to wirelessly collect and then send the EEG data to a remote neurologist and obtain an interpretation for managing the ED patient. A sealed bag contains all the components needed to rapidly obtain an EEG: a headset with integrated electrodes, an analog front-end and analog-to-digital convertor electronics, a digital EEG transmitter and battery module that plugs in to the headset, as well as the electrode gel and an applicator, and operating instructions. (3) eEEG transmission and case management: Via e-mail and electronic instant messaging, the case manager software notifies a network of neurologists who are available to read the EEGs using standard computers of their choosing. The responding neurologist logs in to access the EEG for review and provides a written interpretation. The interpretation is sent back to the ED physician to guide patient care and management. (4) an eEEG system: Plugging in the transmitter/battery module activates electrode impedance testing to determine the appropriate conductive contact to the scalp and give correcting feedback. Patient data are entered using the bar code reader and keypad on the medical tablet personal computer (PC). Once recording is initiated, the EEG is wirelessly transmitted to the medical tablet for display and then to a case management server. The server will also perform real-time automatic seizure detection, setting EEGs with electrophysiological anomalies to high priority for review by one or more remote neurologists.

The novelty in this provisional product is not the EEG, rather it is the eEEG system, which redefines the way the EEG is recorded, using a microEEG in an EEG kit. The eEEG system is poised to fill a glaring need in emergency medicine, namely the need for recording EEGs quickly and affordably in EDs. An eEEG is planned around the microEEG, an inexpensive, miniature, multi-channel, portable wireless system to record the EEG using an electrophysiological recording technology that we describe as digital telemetry (DT). A key innovation is that DT devices reference, amplify, and digitize bioelectric signals at a point very close to the electrodes. The microEEG is inexpensive and miniature because it exploits the billion-dollar market for portable audio applications, which drives chip manufacturers to perfect these circuits by continuously reducing noise, power consumption, size and price while increasing fidelity. DT measures biopotentials with high precision since it digitizes signals on the patient to achieve voltage representations of at least 16 effective bits. The signals are immune to electromagnetic distortion because digitized data are transmitted in the interference-resistant, error-correcting digital Bluetooth protocol. An additional text file discusses the major technical features that this provisional product will address (see Additional file 1).

### Impact of convenient and quick access to eEEG in the ED

Incorporating microEEG into the workup of patients presenting with neurological emergencies should be determined in terms of its impact on the following dimensions: (1) patient-oriented outcomes; (2) cost of care; (3) use of ED resources for managing these patients. Prior work investigated the effects of incorporating an EEG in the workup of patients with mental status in the ED typically by comparing the initial diagnosis of the ED team with the diagnosis at post-examination by a neurologist or post-EEG [4]. In this study, initial abbreviated EEG integration in the ED consistently detected all cases of NCSE. In fact, EEGs performed on an acute, non-elective basis influenced clinical management in selected clinical situations commonly encountered in the ED [3,40]. Utility of acute EEG availability, as defined by its ability to confirm a working diagnosis, rule out a specific diagnosis or help in subsequent patient treatment, could depend on EEG referral diagnosis. The utility of acute EEG recordings was 100% in subjects with a referral diagnosis of SE [3]. In addition to the present evidence on the usefulness of acute EEG availability in early seizure detection and patient management, EEG findings could serve as a prognostic tool for subjects presenting with neurological and non-neurological emergencies [41-43]. It is worth noting that a single EEG with complete generalized suppression in comatose survivors after cardiac arrest indicates no possibility of recovery in the level of consciousness [44].

Although such studies indicate that an emergency EEG has a positive impact on clinical practice, commercial decisions, including hospitals decision to acquire microEEGs, will depend on quantifying the extent that using the device improves patient care and saves ED resources. Optimal use of our proposed device in acute settings dictates that it meets certain technical prerequisites that are exceedingly relevant to ED ambiance. These prerequisites include (1) easy accurate application of electrodes; (2) using an optimally reduced subset of electrodes to minimize electrode application times without compromising the ability to detect cerebral dysfunction; (3) that the microEEG reliably records clinically valid EEGs in the electrically noisy environment of the ED. The above-scrutinized fundamentals are expected to allow for an enhanced approach to various emergencies.

Using the eEEG in the ED particularly in contexts where continuous recording is required necessitates either very frequent review by medical personnel or a system that allows for analysis of the ongoing stream of data. As previously mentioned, sometimes subtle long-term EEG changes correlate with a patient's prognosis and cannot be assessed by visual inspection. In such problematic scenarios, continuous EEG signals must be adapted to other available softwares that could highlight certain features of interest encountered in long-term recording or allow for depicting data in a variety of graphical representations, thereby yielding quantitative measures of long time scales [45].

## Conclusion

Integrating the evidence from various studies characterizing a defined use of EEGs in the ED, we presented an overall product that could account for an unmet need of routine EEG availability in acute care settings, namely the ED. The final state of our proposed apparatus could be diverse in various clinical contexts and should reflect the true requirement of such contexts. Research findings on correlations between neurophysiological parameters and neurological pathologies, and the advancing technologies in data analysis, transmission and display allow for a real enhancement of medical evaluation and management.

## List of abbreviations

**EEG**: Electroencephalogram; **NCSE**: Non-convulsive status epilepticus; **ED**: Emergency department; **AMS**: Alteration in mental status; **sTBI**: Severe traumatic brain injury; **eEEG**: Emergency EEG system; **ICU**: Intensive care unit; **GCSE**: Generalized convulsive status epilepticus; **NCS**: Non-convulsive seizures; **PLED**: Periodic lateralized epileptiform discharges; **PS**: Pseudo-seizures; **PSt**: Pseudo-status epilepticus; **AEDs**: Anti-epileptic drug; **PC**: Personal computer; **DT**: Digital telemetry.

## Competing interests

All authors are collaborating with Biosignal Group Inc. in a study funded by the National Institutes of Health. SGAB, AO, and AF receive salary support from or have financial stakes in Biosignal Group Inc. SZ receives salary support from the NIH grant through Downstate Medical Center.

## Authors' contributions

SGAB and SZ performed the literature search and drafted the manuscript. AO and AF provided the technical characteristics of the proposed device and made significant contributions in editing the manuscript. All authors read and approved the final manuscript.

## Supplementary Material

Additional file 1 **Technical Features of the Provisional Device**. Additional file summarizes the technical aspects of the provisional device including noise reduction, data transmission and signal processing. Click here for file
