# Supplementary material for: The new wave: time to bring EEG to the emergency department
Source: Int J Emerg Med. 2011 Jun 24;4:36. doi: 10.1186/1865-1380-4-36 (PMC3145557; doi:10.1186/1865-1380-4-36)
Supplement: Additional file 1 — Technical Features of the Provisional Device. Additional file summarizes the technical aspects of the provisional device including noise reduction, data transmission and signal processing. [file 1865-1380-4-36-S1.PDF]

The major technical features that this provisional product addresses include the following:

1- DT communication has no constraints on the origin of the digital data it transmits.

2- Data can be arranged in any way by the processor.

3- Digital signal processing can occur at the signal source before the data are transmitted from the patient.

4- DT can assess signal fidelity, avoiding signal distortion during transmission.

5- Noise immunity could be due to a fundamental difference between microEEG and other conventional EEG recording amplifiers.

6- microEEG's wireless data transmission uses Bluetooth technology and could be applicable up to a distance of 10 meters.

In conclusion, one can draw the following summary of the technological aspects that characterize this proposed device: (i) microEEG device references, amplifies, and digitizes the signals at a point very close to the electrodes; (ii) microEEG device can acquire signals with very few electronic components per channel; (iii) microEEG device can transmit the digital signals either wirelessly or optically to a PC using the Bluetooth protocol.
